# Association between Comorbidities and Progression of Transvalvular Pressure Gradients in Patients with Moderate and Severe Aortic Valve Stenosis

**DOI:** 10.1155/2018/3713897

**Published:** 2018-11-11

**Authors:** Tim Salinger, Kai Hu, Dan Liu, Scharoch Taleh, Sebastian Herrmann, Daniel Oder, Daniel Gensler, Jonas Müntze, Georg Ertl, Kristina Lorenz, Stefan Frantz, Frank Weidemann, Peter Nordbeck

**Affiliations:** ^1^Department of Internal Medicine I, University Hospital Würzburg, Würzburg, Germany; ^2^Comprehensive Heart Failure Center (CHFC), University of Würzburg, Würzburg, Germany; ^3^Magnetic Resonance and X-ray Imaging MRB, Development Center X-ray Technology EZRT, Fraunhofer Institute for Integrated Circuits IIS, Würzburg, Germany; ^4^Department of Pharmacology, Institute of Pharmacology and Toxicology, Würzburg, Germany; ^5^Leibniz-Institut für Analytische Wissenschaften – ISAS, University Duisburg-Essen, Dortmund, Germany; ^6^Medizinische Klinik I des Klinikum Vest, Recklinghausen, Germany

## Abstract

**Background:**

Fast progression of the transaortic mean gradient (*P*_mean_) is relevant for clinical decision making of valve replacement in patients with moderate and severe aortic stenosis (AS) patients. However, there is currently little knowledge regarding the determinants affecting progression of transvalvular gradient in AS patients.

**Methods:**

This monocentric retrospective study included consecutive patients presenting with at least two transthoracic echocardiography examinations covering a time interval of one year or more between April 2006 and February 2016 and diagnosed as moderate or severe aortic stenosis at the final echocardiographic examination. Laboratory parameters, medication, and prevalence of eight known cardiac comorbidities and risk factors (hypertension, diabetes, coronary heart disease, peripheral artery occlusive disease, cerebrovascular disease, renal dysfunction, body mass index ≥30 Kg/m^2^, and history of smoking) were analyzed. Patients were divided into slow (*P*_mean_ < 5 mmHg/year) or fast (*P*_mean_ ≥ 5 mmHg/year) progression groups.

**Results:**

A total of 402 patients (mean age 78 ± 9.4 years, 58% males) were included in the study. Mean follow-up duration was 3.4 ± 1.9 years. The average number of cardiac comorbidities and risk factors was 3.1 ± 1.6. Average number of cardiac comorbidities and risk factors was higher in patients in slow progression group than in fast progression group (3.3 ± 1.5 vs 2.9 ± 1.7; *P*=0.036). Patients in slow progression group had more often coronary heart disease (49.2% vs 33.6%; *P*=0.003) compared to patients in fast progression group. LDL-cholesterol values were lower in the slow progression group (100 ± 32.6 mg/dl vs 110.8 ± 36.6 mg/dl; *P*=0.005).

**Conclusion:**

These findings suggest that disease progression of aortic valve stenosis is faster in patients with fewer cardiac comorbidities and risk factors, especially if they do not have coronary heart disease. Further prospective studies are warranted to investigate the outcome of patients with slow versus fast progression of transvalvular gradient with regards to comorbidities and risk factors.

## 1. Introduction

Stenosis of the aortic valve (AS) is a common clinical finding, especially in elderly patients. In population studies, the prevalence of moderate or severe AS has been reported as about 3% of subjects ≥75 years by Nkomo et al. [1] and Lindroos et al. [2] reported that AS prevalence was 2.9% among people aged 75 to 85 years (aortic valve opening area (AVA) ≤0.8 cm^2^) or 4.8% (AVA ≤1.2 cm^2^) depending on the AVA. Prevalence of severe AS increased to 5.9%, and the 3-year mortality was about 40% in a population aged ≥80 years [3]. Studies also showed that AS was complicated by various cardiac and noncardiac comorbidities in elderly populations [4, 5]. Previous studies demonstrated the relevant prognostic impact of many factors including low systolic blood pressure [6], depressed systemic arterial compliance [7], left ventricular ejection fraction and mean transvalvular gradient [8], and the outcome with multiple comorbidities and cardiac risk factors was poor in AS patients undergoing surgical valve replacement [9].

Comprehensive epidemiological data about clinical characteristics of patients with valvular heart disease were described in the *Euro Heart Survey on Valvular heart disease* [10]. It is known that common origins existed in the pathophysiology of AS and coronary heart disease [11, 12], and some dissimilarities of pathophysiology were also found between the two diseases [13]. Boudoulas et al. demonstrated a high incidence of coronary atherosclerosis in patients with severe AS undergoing surgical aortic valve replacement [14]. Aronow et al. investigated the impact of several cardiovascular comorbidities on mild AS in a retrospective analysis including 180 patients aged ≥60 years and reported male gender, cigarette smoking, systemic hypertension, diabetes mellitus, high low-density lipoprotein cholesterol (LDL-C), and low high-density lipoprotein cholesterol (HDL-C) were associated with fast progression of AS [15]. However, data regarding the impact of cardiac comorbidities and cardiovascular risk factors on the progression of the mean transaortic valve pressure gradient (*P*_mean_) in patients with moderate or severe AS remain scarce in the literature until now. In this context, especially the role of LDL-C and impact of statin therapy on aortic valve calcification have become the subject of controversial debates [16–18]. The European Society of Cardiology guidelines on the diagnosis and therapy of valvular heart diseases list rapid progression of AS assessed by echocardiography as a prognostic indicator justifying early aortic valve replacement in asymptomatic patients, but also fall short in providing any potential pathophysiological or mechanistic insights [19]. Therefore, we investigated potential associations between the progression of AS assessed by *P*_mean_ from serial transthoracic echocardiography and the number of cardiac comorbidities and risk factors in patients with moderate or severe AS in the current study.

## 2. Methods

### 2.1. Study Population and Protocol

Adult patients who were diagnosed for acquired moderate or severe AS at final echocardiography and underwent at least two transthoracic echocardiography examinations covering a time interval of one year or more between April 2006 and February 2016 in our department were included in this study. All patients underwent physical examination, transthoracic echocardiography by an experienced examiner, and routine blood testing. Furthermore, blood pressure, body mass index, and medical history at baseline were obtained. Patients were divided into two groups according to the average *P*_mean_ progression of the cohort (4.52 mmHg/year)—slow progression group: annual *P*_mean_ progression <5 mmHg/year and fast progression group: annual *P*_mean_ progression ≥5 mmHg/year.

We then compared the prevalence of eight known cardiac comorbidities and risk factors (hypertension, diabetes, coronary heart disease, peripheral artery occlusive disease, cerebrovascular disease, renal dysfunction, body mass index (BMI) higher than 30 kg/m^2^, and history of smoking) between the two groups. Furthermore, several laboratory values such as LDL-C, HDL-C, hemoglobin A1c (HbA1c), hemoglobin, creatinine, and C-reactive protein (CRP) were compared between the two groups.

### 2.2. Echocardiographic Measurements

A complete echocardiographic examination was performed at baseline and follow-up using Vingmed Vivid 7 or E9 (General Electric, Horten, Norway). Standard measurements were performed according to the ASE guidelines [12]. LV ejection fraction was measured using the biplane Simpson method in the apical 4- and 2-chamber views. The diagnosis and classification of AS were performed according to EAE/ASE recommendations [13]. The outer edge of the AS jet velocity curve in continuous-wave Doppler imaging was traced to obtain maximum AS jet velocity and maximal and mean transaortic pressure gradients (*P*_max_ and *P*_mean_).

### 2.3. Statistical Analysis

Statistical analysis was performed using SPSS V 24 (IBM Corp. Armonk, NY, United States). Continuous variables are presented as mean ± standard deviation (SD). Continuous variables of demographic, disease, and echocardiographic characteristics between groups (i.e., nonprogression vs. progression; male vs. female; <80 years vs. ≥80 years) were, respectively, compared using unpaired Student's *t*-test. Categorical variables were expressed as percentages, and differences between groups were compared using a chi-squared test or Fisher's exact test, as appropriate. Multivariable logistic regression analysis was conducted to identify independent determinants of faster progression in *P*_mean_ (≥5 mmHg/year) in this cohort, separately adjusted for age, sex, age and sex, as well as age, sex, and left ventricular ejection fraction (LVEF). Odds ratio (OR) with 95% confidence interval (CI) was used to assess the independent determinants. A two-tailed *P* < 0.05 was used to indicate statistical significance.

## 3. Results

A total of 402 consecutive patients (mean age: 78 ± 9.4 years at follow-up echocardiography, 58% males) were included in this study. There are no rheumatic valve disease in this cohort. The average number of cardiac comorbidities and risk factors in the cohort was 3.1 ± 1.6.  Table 1 shows clinical characteristics of the total cohort at baseline.

As shown in Table 2, prevalence of coronary artery disease and history of myocardial infarction at baseline was significantly lower in the fast progression group than in the slow progression group (both *P* < 0.05). In addition, there was a trend for lower prevalence of peripheral vascular disease and renal dysfunction at baseline in the fast progression group compared to the slow progression group (*P*=0.08 and 0.09). At follow-up echocardiography, besides lower prevalence of coronary artery disease and history of myocardial infarction, incidence of diabetes was also lower in the fast progression group as compared to the slow progression group. Annual progression in AV *V*_max_ and AVA VTI were also slow as *P*_mean_ in the slow progression group as compared to the fast progression group. Moreover, patients in the slow progression group of *P*_mean_ had significantly more overall cardiac comorbidities and risk factors as compared to the fast progression group (3.3 ± 1.5 vs. 2.9 ± 1.7; *P*=0.036). Both LDL-C and HDL-C values were considerably higher, whereas CRP values were lower in the fast progression group as compared to the slow progression group (Table 3). Percent of patients treated with statins at the time of follow-up echocardiography was similar (fast progression: 55% vs. slow progression: 61.1%; *P*=0.270).

Echocardiographic characteristics of the two groups at baseline and follow-up are shown in Table 4. Baseline parameters were largely similar between slow and fast progression groups except lower LVEF and maximal transaortic velocity (*V*_max_) values in the slow progression group than in the fast progression group. Left ventricular septal (IVSd) and posterior wall thickness (LVPWd), LVEF, AV *V*_max_, AV *P*_max_, and AV *P*_mean_ were significantly higher while aortic valve area calculated by the velocity time integral (AVA VTI) value was significantly lower in the fast progression group than in the slow progression group at follow-up echocardiography. Compared to baseline values, LVPWd, LVEF, and AVA VTI were reduced, while left atrial diameter, AV *V*_max_, AV *P*_max_, and AV *P*_mean_ were increased in the slow progression group. AVA VTI was significantly reduced, while AV *V*_max_, AV *P*_max_, and AV *P*_mean_ were significantly increased in the fast progression group.

Odds ratio of cardiac comorbidities and risk factors and laboratory parameters at baseline associated with fast *P*_mean_ progression are shown in Table 5. Univariable logistic regression analysis showed that higher LVEF, lower prevalence of coronary heart disease and myocardial infarction, lower number of comorbidities, higher LDL-C, and HDL-C values were associated with a fast *P*_mean_ progression over time. After adjustment for age, sex, and LVEF, coronary heart disease, history of myocardial infarction, the number of comorbidities, and LDL-C at baseline remained as independent risk factors associated with *P*_mean_ progression.

Supplementary Tables 1 and 2 demonstrate the associations between cardiac comorbidities and risk factors at baseline and follow-up as well as laboratory parameters and fast *P*_mean_ progression, after adjustment for age, sex, and age plus sex, respectively. Diabetes at follow-up, coronary heart disease, and history of myocardial infarction at baseline and follow-up remained as risk factors of slow progression of *P*_mean_, and absence of these factors serve as factors indicating a fast progression of *P*_mean_. Higher average number of comorbidities and lower values of LDL-C and HDL-C remained as risk factors of slow progression of *P*_mean_ after adjusted for age and sex.

## 4. Discussion

This retrospective study reveals a context between progressions of the mean transaortic valve pressure gradient (*P*_mean_) in patients with AS and the prevalence of cardiac comorbidities and risk factors. Based on the study findings, *P*_mean_ progress is considerably slower in patients with a higher prevalence of cardiac comorbidities and risk factors as compared to those with less prevalence of these factors. Under closer examination, our results indicate that prevalence of coronary heart disease might indeed be the comorbidity with the highest impact on *P*_mean_ progression, but also other comorbidities such as renal dysfunction and peripheral artery occlusive disease tended to be less common in aortic stenosis patients with fast *P*_mean_ progression.

The underlying reasons for above finding might be multiple. We speculate that myocardial infarction with subsequent cardiac fibrosis could lead to the inability of building up the pressure within left ventricle, which would be responsible for reduced *P*_mean_. Consequently, a relative lower LVEF might also be associated with slower progression of *P*_mean_ (Table 4). Although LVEF at baseline and at follow-up was statistically lower in the slow progression group than in the fast progression group (*P*=0.02 and *P*=0.002), the difference is rather negligible: 2.9% at baseline and 3.8% at follow-up, and the mean EF value was above 50% at both the slow and fast progression groups. Moreover, the prevalence of LVEF<50% at baseline was 18.7% in the slow progression group and 14.3% in the fast progression group (*P*=0.260), Therefore, it is unlikely that the *P*_mean_ progression difference was affected by the LVEF values in this study.

No relevant differences were found regarding obesity, hypertension, or nicotine abuse. These results fit to recently published findings indicating a relevant prevalence of coronary artery disease in respective cohorts with aortic stenosis [20]. In contrast to the results from our current study, Rosenhek et al. reported a faster increase in aortic stenosis progression in patients with coronary artery disease [21]. However, this study investigated a cohort with mild and moderate aortic stenosis with patients considerably younger than those in our study, whereas our study population included older patients on average with moderate and severe stenosis.

As aortic stenosis and coronary heart disease might share some pathophysiological background [11, 12], they might also share similar cardiac comorbidities and risk factors [22]. In our study, almost half of the aortic stenosis patients had coronary artery disease, but there was no correlation between a higher incidence of coronary heart disease and faster progress of *P*_mean_. Nevertheless, we found a slower progression of *P*_mean_ in patients with coronary heart disease as well as in patients with other cardiac comorbidities and risk factors, independent of age, sex, and LVEF. Reduced myocardial contracting power and enhance myocardial fibrosis, reduced myocardial microcirculation, and myocardial-vascular incompetence might be responsible for the observe phenomenon. However, these cannot be verified on the basis of the current data.

In our cohort, patients with fast progress of *P*_mean_ had higher levels of LDL-C, independent of age, sex, and LVEF. In line with these findings, former studies also revealed higher values of LDL-C to be a risk factor for AS [23] although no benefit from statin therapy on valve function and calcification could be found so far [24, 25]. Paradoxically, LDL-C was an independent risk factor for faster progress of *P*_mean_ in our cohort in the context of similar statins application between the two groups, whereas prevalence of coronary artery disease was higher in the slow progression group. This finding is compatible with former studies showing no impact of statins on AS progression [25, 26]. Recently, Thaigo et al. presented a review showing lower values of *P*_mean_ in patients who were under statin therapy, but they also agree the analyzed studies did not have high quality and concluded that the role of statin therapy in AS remained uncertain [27]. CRP values were also high in the slow progression group indicating increased inflammatory stress in these patients; this could be fairly explained by the increased inflammation status and higher comorbidities in these patients.

It is to note that the diagnosis of definite bicuspid aortic valve was difficult based on transthoracic echocardiography imaging, we thus could not define the impact of bicuspid valve on the *P*_mean_ progression in this patient cohort.

## 5. Conclusions

The results of the current study imply that patients with moderate or severe aortic stenosis and a high prevalence of cardiac comorbidities and risk factors, especially history of myocardial infarction and coronary heart disease, diabetes, generally show slower progression of the mean aortic valve pressure gradient compared to patients with a low prevalence of cardiac comorbidities and risk factors. Higher levels of LDL-C are a risk factor for fast progression of *P*_mean_, while higher CRP is linked with slow *P*_mean_ progression, indicating a strong correlation between prevalence of cardiac comorbidities and risk factors and inflammation stress. Future studies are warranted to explore the outcome differences between aortic stenosis patients with slow and fast *P*_mean_ progression.

## Figures and Tables

**Table 1:** Characteristics of the cohort at baseline.

	Total cohort
Number	402
Male gender (*n* (%))	233 (58)
Age (years)	74.1 ± 9.8
BMI (kg/m^2^)	28.1 ± 5.0
History of smoking (%)	36.3
Diabetes (%)	35.8
Coronary heart disease (%)	43.8
Myocardial infarction (%)	22.0
Hypertension (%)	87.1
Renal dysfunction (%)	53.3
Peripheral artery occlusive disease (%)	23.1
Cerebrovascular disease (%)	14.7
Echo LVEF (%)	53.8 ± 11.5
Echo *P*_mean_ (mmHg)	25.01 ± 14.48

BMI, body mass index; LVEF, left ventricular ejection fraction; *P*_mean_, mean transaortic pressure gradient.

**Table 2:** Comorbidities of patients with slow or fast progression of mean transaortic pressure gradient at baseline and follow-up echocardiography.

	Total cohort		Slow progression (*P*_mean_ < 5 mmHg/year)		Fast Progression (*P*_mean_ ≥ 5 mmHg/year)		*P* value
	*N*=402	%	*N*=262	%	*N*=140	%	
*Sex*							0.98
Male	233	58.0	152	58.0	81	57.9	
Female	169	42.0	110	42.0	59	42.1	
*Comorbidities at baseline*							
BMI≥30 Kg/m^2^	89	29.4	55	28.1	34	31.8	0.51
History of smoking	146	36.3	96	36.6	50	35.7	0.91
Diabetes	144	35.8	101	38.5	43	30.7	0.13
Coronary heart disease	176	43.8	129	49.2	47	33.6	**0.003**
Myocardial infarction	88	21.9	67	25.6	21	15.0	**0.016**
Hypertension	350	87.1	230	87.8	120	85.7	0.64
Renal dysfunction	210	53.3	146	56.6	64	47.1	0.09
Peripheral artery occlusive disease	93	23.1	68	26	25	17.9	0.08
Cerebrovascular disease	59	14.7	34	13	25	17.9	0.24
*Comorbidities at follow-up*							
BMI≥30 Kg/m^2^	145	38.7	96	39.3	49	37.4	0.70
History of smoking	147	36.6	97	37	50	35.7	0.80
Diabetes	161	40.0	115	43.9	46	32.9	**0.033**
Coronary heart disease	228	56.9	159	60.9	69	49.3	**0.027**
Myocardial infarction	104	25.9	81	30.9	23	16.4	**0.002**
Hypertension	368	91.5	239	91.2	129	92.1	0.85
Renal dysfunction	281	69.9	188	71.8	93	66.4	0.30
Peripheral artery occlusive disease	141	35.1	93	35.5	48	34.3	0.83
Cerebrovascular disease	79	19.7	44	16.8	35	25.0	0.06
History of statin-therapy	244	65.4	149	61.1	71	55.0	0.27

BMI, body mass index; *P*_mean_, mean transaortic pressure gradient.

**Table 3:** Clinical and laboratory characteristics.

	Total cohort	Slow progression (*P*_mean_ < 5 mmHg/year)	Fast Progression (*P*_mean_ ≥ 5 mmHg/year)	*P* value
	*N*=402	*N*=262	*N*=140	
Age at follow-up (years)	77.7 ± 9.4	77.5 ± 9.8	78 ± 8.6	0.65
Number of comorbidities	3.1 ± 1.6	3.3 ± 1.5	2.9 ± 1.7	**0.036**
Annual progression in AV *P*_mean_ (mmHg/year)	4.5 ± 4.6	1.8 ± 1.6	9.5 ± 4.2	**<0.001**
Annual progression in AV *V*_max_ (m/s/year)	0.08 ± 0.18	0.01 ± 0.12	0.22 ± 0.18	**<0.001**
Annual progression in AVA VTI (cm^2^/year)	−0.14 ± 0.14	−0.12 ± 0.13	−0.18 ± 0.16	**<0.001**
Baseline LVEF <50% (n (%))	69 (17.2%)	49 (18.7%)	20 (14.3%)	0.26
LDL-cholesterol (mg/dl)	103.8 ± 34.4	100 ± 32.6	110.8 ± 36.6	**0.005**
HDL-cholesterol (mg/dl)	52.0 ± 16.7	50.6 ± 16.3	54.7 ± 17.1	**0.025**
HbA1c (%)	6.3 ± 1	6.34 ± 1	6.28 ± 1	0.65
Hemoglobin (g/dl)	12.9 ± 3.8	12.7 ± 2.9	13.1 ± 5.1	0.32
C-reactive protein (mg/dl)	1.7 ± 2.4	1.9 ± 2.7	1.4 ± 1.9	**0.025**
Creatinine (mg/dl)	1.5 ± 1.2	1.5 ± 1.1	1.4 ± 1.3	0.72

AV, aortic valve; *P*_mean_, mean transaortic pressure gradient; *V*_max_, maximum velocity by continuous-wave Doppler; AVA VTI, aortic valve area calculated by the velocity time integral; LVEF, left ventricular ejection fraction; LDL, low-density lipoprotein; HDL, high-density lipoprotein; HbA1c, hemoglobin A1c.

**Table 4:** Echocardiographic characteristics between baseline and follow-up.

	Total cohort	Slow progression (*P*_mean_ < 5 mmHg/year)	Fast Progression (*P*_mean_ ≥ 5 mmHg/year)	*P* value
	*N*=402	*N*=262	*N*=140	
*LVEDD (mm)*				
Baseline	47.9 ± 8.6	48.4 ± 8.9	47.0 ± 7.9	0.11
Follow-up	47.86 ± 8.5	48.2 ± 8.9	47.2 ± 7.7	0.23

*IVSd (mm)*				
Baseline	11.5 ± 2.2	11.5 ± 2.2	11.6 ± 2.2	0.58
Follow-up	11.4 ± 2.3	11.2 ± 2.5	11.9 ± 1.9	**0.001**

*LVPWd (mm)*				
Baseline	11.2 ± 2.0	11.2 ± 2.0	11.3 ± 2.0	0.43
Follow-up	11.1 ± 2.0	10.9 ± 2.0^*∗*^	11.7 ± 1.8	**<0.001**

*LVEF (%)*				
Baseline	53.8 ± 11.5	52.8 ± 11.5	55.7 ± 11.2	**0.020**
Follow-up	51.4 ± 12.4^*∗*^	50.1 ± 13.0^*∗*^	53.9 ± 10.7	**0.002**

*LAD (mm)*				
Baseline	41.5 ± 6.6	41.4 ± 6.9	41.8 ± 6.0	0.62
Follow-up	42.7 ± 6.6^*∗*^	42.7 ± 7.0^*∗*^	42.79 ± 5.9	0.86

*AVA VTI (cm* ^*2*^)				
Baseline	1.3 ± 0.5	1.4 ± 0.4	1.3 ± 0.5	0.09
Follow-up	0.9 ± 0.3^*∗*^	1.0 ± 0.2^*∗*^	0.8 ± 0.2^*∗*^	**<0.001**

*AV V*_*max*_ * (m/s)*				
Baseline	3.1 ± 0.9	3.0 ± 0.9	3.2 ± 0.8	**0.03**
Follow-up	3.7 ± 0.9^*∗*^	3.4 ± 0.8^*∗*^	4.3 ± 0.7^*∗*^	**<0.001**

*AV P*_*max*_ * (mmHg)*				
Baseline	40.9 ± 22.5	39.75 ± 23.3	43.2 ± 20.72	0.14
Follow-up	57.6 ± 25.9^*∗*^	47.4 ± 21.1^*∗*^	76.6 ± 23.3^*∗*^	**<0.001**

*AV P*_*mean*_ * (mmHg)*				
Baseline	25.0 ± 14.5	24.3 ± 15.1	26.3 ± 13.1	0.20
Follow-up	37.0 ± 17.5^*∗*^	29.8 ± 13.8^*∗*^	50.3 ± 15.9^*∗*^	**<0.001**

^*∗*^ *P* < 0.05 vs. baseline. LVEDD, left ventricular end-diastolic dimension; IVSd, intraventricular septum thickness; LVPWd, left ventricular posterior wall thickness; LVEF, left ventricular ejection fraction; LAD, left atrial dimension; AVA VTI, aortic valve area calculated by the velocity time integral; AV, aortic valve; *P*_max_, maximal transaortic pressure gradient; *P*_mean_, mean transaortic pressure gradient.

**Table 5:** Odds ratio of cardiac comorbidities and risk factors and laboratory parameters at baseline associated with fast *P*_mean_ progression.

	Crude OR (95% CI)	*P* value	Age and sex adjusted OR (95% CI)	*P* value	Age, sex, and LVEF adjusted OR (95% CI)	*P* value
Age	1.005 (0.983–1.028)	0.644	—	—	—	—
Male gender	0.994 (0.656–1.505)	0.976	—	—	—	—
LVEF	1.022 (1.003–1.042)	**0.023**	1.023 (1.003–1.043)	**0.023**	—	—
BMI ≥ 30 kg/m^2^	1.194 (0.715–1.993)	0.498	—	—	—	—
History of smoking	0.961 (0.627–1.473)	0.854	—	—	—	—
Diabetes	0.707 (0.457–1.094)	0.119	—	—	—	—
Coronary heart disease	0.521 (0.340–0.798)	**0.003**	0.487 (0.312–0.760)	**0.002**	0.496 (0.317–0.777)	**0.002**
Myocardial infarction	0.514 (0.299–0.882)	**0.016**	0.494 (0.285–0.856)	**0.012**	0.532 (0.304–0.931)	**0.027**
Hypertension	0.835 (0.458–1.522)	0.556	—	—	—	—
Renal dysfunction	0.682 (0.449–1.035)	0.072	0.655 (0.428–1.002)	0.051	0.692 (0.450–1.064)	0.093
Peripheral artery occlusive disease	0.620 (0.371–1.036)	0.068	0.609 (0.363–1.022)	0.061	0.610 (0.362–1.027)	0.063
Cerebrovascular disease	1.458 (0.830–2.560)	0.189	—	—	—	—
Number of comorbidities	0.869 (0.761–0.991)	**0.037**	0.852 (0.741–0.980)	**0.025**	0.863 (0.750–0.994)	**0.041**
LDL-cholesterol	1.009 (1.002–1.016)	**0.009**	1.010 (1.003–1.017)	**0.005**	1.010 (1.003–1.017)	**0.006**
HDL-cholesterol	1.015 (1.001–1.029)	**0.034**	1.015 (1.001–1.029)	**0.042**	1.014 (1.000–1.028)	0.051
HbA1c	0.969 (0.764–1.229)	0.793	—	—	—	—
Hemoglobin	1.016 (0.985–1.047)	0.326	—	—	—	—
C-reactive protein	0.952 (0.874–1.036)	0.254	—	—	—	—
Creatinine	1.001 (0.799–1.253)	0.996	—	—	—	—

*P*_mean_, mean transaortic pressure gradient; OR, odds ratio; CI, confidence interval; LDL, low-density lipoprotein; HDL, high-density lipoprotein; HbA1c, hemoglobin A1c.

## Data Availability

All data generated or analyzed during this study are available from the corresponding author on reasonable request.

## Supplementary Materials

Supplementary Materials Supplementary tables 1 and 2 demonstrate the associations between cardiac comorbidities and risk factors at baseline and follow-up as well as laboratory parameters and fast *P*_mean_ progression, after adjustment for age, sex, and age plus sex, respectively. Diabetes at follow-up, coronary heart disease, and history of myocardial infarction at baseline and follow-up remained as risk factor of slow progression of *P*_mean_ and absence of these factors serve as factors indicating a fast progression of *P*_mean_. Higher average number of comorbidities and lower values of LDL-C and HDL-C remained as risk factors of slow progression of *P*_mean_ after adjusted for age and sex.
