# Supplementary material for: Association between Comorbidities and Progression of Transvalvular Pressure Gradients in Patients with Moderate and Severe Aortic Valve Stenosis
Source: Cardiol Res Pract. 2018 Nov 11;2018:3713897. doi: 10.1155/2018/3713897 (PMC6252229; doi:10.1155/2018/3713897)
Supplement: Supplementary Materials — Supplementary tables 1 and 2 demonstrate the associations between cardiac comorbidities and risk factors at baseline and follow-up as well as laboratory parameters and fast P mean progression, after adjustment for age, sex, and age plus sex, respectively. Diabetes at follow-up, coronary heart disease, and history of myocardial infarction at baseline and follow-up remained as risk factor of slow progression of P mean and absence of these factors serve as factors indicating a fast progression of P mean. Higher average number of comorbidities and lower values of LDL-C and HDL-C remained as risk factors of slow progression of P mean after adjusted for age and sex. [file 3713897.f1.pdf]

Supplementary table 1 Odds ratio of cardiac comorbidities and risk factors at baseline and follow-up associated with fast Pmean progression after adjusted for age and sex

	**OR adjusted for age (95% CI)**	**P value**	**OR adjusted for sex (95% CI)**	**P value**	**OR adjusted for age and sex (95% CI)**	**P value**
Baseline BMI≥30 Kg/m²	1.260 (0.749-2.121)	0.383	1.196 (0.716-1.997)	0.495	1.260 (0.749-2.120)	0.385
Follow-up BMI≥30 Kg/m²	0.928 (0.599-1.439)	0.740	0.918 (0.592-1.423)	0.918	0.925 (0.596-1.434)	0.726
Baseline history of smoking	0.975 (0.633-1.502)	0.908	0.955 (0.593-1.538)	0.849	0.965 (0.598-1.557)	0.883
Follow-up history of smoking	0.958 (0.622-1.475)	0.845	0.936 (0.584-1.501)	0.785	0.945 (0.589-1.028)	0.820
Baseline diabetes	0.707 (0.457-1.094)	0.119	0.706 (0.456- 1.094)	0.119	0.706 (0.456-1.093)	0.119
Follow-up diabetes	0.626 (0.407-0.961)	**0.032**	0.626 (0.407-0.961)	**0.032**	0.626 (0.407-0.961)	**0.032**
Baseline coronary heart disease	0.512 (0.333-0.786)	**0.002**	0.502 (0.324-0.779)	**0.002**	0.487 (0.312-0.760)	**0.002**
Follow-up coronary heart disease	0.611 (0.403-0.928)	**0.021**	0.0606 (0.395-0.931)	**0.022**	0.587 (0.379-0.907)	**0.016**
Baseline myocardial infarction	0.505 (0.294-0.869)	**0.014**	0.506 (0.293-0.874)	**0.015**	0.494 (0.285-0.856)	**0.012**
Follow-up myocardial infarction	0.430 (0.255-0.724)	**0.002**	0.430 (0.254-0.727)	**0.002**	0.416 (0.245-0.707)	**0.001**
Baseline hypertension	0.807 (0.437- 1.487)	0.491	0.835 (0.458-1.522)	0.556	0.806 (0.437-1.487)	0.490
Follow-up hypertension	1.088 (0.504-2.351)	0.829	1.129 (0.533-2.388)	0.750	1.088 (0.504-2.351)	0.830
Baseline renal dysfunction	0.661 (0.433-1.008)	0.054	0.680 (0.448- 1.033)	0.071	0.655 (0.428-1.002)	0.051
Follow-up renal dysfunction	0.749 (0.475-1.180)	0.212	0.779 (0.5-1.212)	0.268	0.747 (0.473-1.179)	0.210
Baseline peripheral artery occlusive disease	0.614 (0.367-1.027)	0.063	0.617 (0.368-1.034)	0.067	0.609 (0.363-1.022)	0.061
Follow-up peripheral artery occlusive disease	0.945 (0.614-1.454)	0.795	0.948 (0.611-1.469)	0.810	0.941 (0.606-1.46)	0.786
Baseline cerebrovascular disease	1.453 (0.827-2.552)	0.194	1.458 (0.830-2.560)	0.190	1.453 (0.827-2.553)	0.193
Follow-up cerebrovascular disease	1.647 (0.997-2.719)	0.051	1.652 (1.000-2.727)	**0.050**	1.647 (0.998-2.721)	0.051

Pmean, mean trans-aortic pressure gradient; OR, odds ratio; CI, confidence interval; BMI, body mass index

Supplementary table 2 Odds ratio of laboratory parameters associated with fast Pmean progression after adjusted for age and sex

	OR adjusted for age (95% CI)	P value	OR adjusted for sex (95% CI)	P value	OR adjusted for age and sex (95% CI)	P value
Number of comorbidities	0.866 (0.759-0.989)	**0.034**	0.858 (0.747-0.985)	**0.029**	0.852 (0.741-0.980)	**0.025**
LDL- Cholesterol	1.010 (1.003-1.016)	**0.004**	1.009 (1.003-1.016)	**0.007**	1.010 (1.003-1.016)	**0.005**
HDL-Cholesterol	1.015 (1.002-1.028)	**0.027**	1.015 (1.001-1.028)	**0.034**	1.015 (1.001-1.028)	**0.035**
HbA1c	0.946 (0.756-1.183)	0.624	0.946 (0.756-1.183)	0.626	0.941 (0.751-1.178)	0.594
Hemoglobin	1.028 (0.972-1.087)	0.335	1.027 (0.971-1.086)	0.346	1.028 (0.972-1.088)	0.332
C-reactive protein	0.897 (0.804-1.001)	0.052	0.896 (0.803-1)	**0.050**	0.897 (0.804-1.001)	0.053
Creatinine	0.971 (0.808-1.166)	0.750	0.967 (0.803-1.164)	0.719	0.970 (0.805-1.169)	0.752

Pmean, mean trans-aortic pressure gradient; OR, odds ratio; CI, confidence interval; LDL, low-density lipoprotein; HDL, high-density lipoprotein; HbA1c, hemoglobin A1c
